# An open label phase II study evaluating first-line *EGFR* tyrosine kinase inhibitor erlotinib in non-small cell lung cancer patients with tumors showing high *EGFR* gene copy number

**DOI:** 10.18632/oncotarget.13793

**Published:** 2016-12-04

**Authors:** Ewa Szutowicz-Zielinska, Krzysztof Konopa, Anna Kowalczyk, Malgorzata Suszko-Kazarnowicz, Renata Duchnowska, Aleksandra Szczesna, Magdalena Ratajska, Aleksander Sowa, Janusz Limon, Wojciech Biernat, Tomasz Burzykowski, Jacek Jassem, Rafal Dziadziuszko

**Affiliations:** ^1^ Department of Oncology and Radiotherapy, Medical University of Gdansk, Poland; ^2^ Department of Oncology, The Centre for Pulmonary Diseases Olsztyn, Poland; ^3^ Department of Oncology, Military Institute of Medicine, Warsaw, Poland; ^4^ Department of Lung Diseases, Mazovian Centre for Treatment of Lung Diseases and Tuberculosis, Otwock, Poland; ^5^ Department of Biology and Genetics, Medical University of Gdansk, Poland; ^6^ Roche, Poland; ^7^ Department of Pathomorphology, Medical University of Gdansk, Poland; ^8^ Interuniversity Institute of Biostatistics and Statistical Bioinformatics, Hasselt University, Diepenbeek, Belgium

**Keywords:** non-small cell lung cancer, epidermal growth factor receptor, gene copy number, erlotinib

## Abstract

**Background:**

First-line treatment with epidermal growth factor receptor (EGFR) inhibitors in NSCLC is effective in patients with activating *EGFR* mutations. The activity of erlotinib in patients harboring high *EGFR* gene copy number has been considered debatable.

**Patients and Methods:**

A multicenter, open-label, single-arm phase II clinical trial was performed to test the efficacy of erlotinib in the first-line treatment of NSCLC patients harboring high *EGFR* gene copy number defined as =4 copies in =40% of cells.

**Findings:**

Between December 2007 and April 2011, tumor samples from 149 subjects were screened for *EGFR* gene copy number by fluorescence in-situ hybridization (FISH), Out of 49 patients with positive *EGFR* FISH test, 45 were treated with erlotinib. Median PFS in the intent-to-treat population was 3.3 months (95%CI: 1.83.9 months), and median overall survival was 7.9 months (95% CI: 5.112.6 months). Toxicity profile of erlotinib was consistent with its known safety profile. The trial was stopped prematurely at 63% of originally planned sample size due to accumulating evidence that *EGFR* gene copy number should not be used to select NSCLC patients to first-line therapy with EGFR TKI. Data on erlotinib efficacy according to *EGFR*, *KRAS* and *BRAF* mutations are additionally presented.

**Interpretation:**

This trial argues against using high gene copy number for selection of NSCLC patients to first-line therapy with EGFR TKIs. The study adds to the discussion on efficacy of other targeted agents in patients with target gene amplified tumors.

## INTRODUCTION

First and second generation EGFR inhibitors, erlotinib, gefitinib and afatinib, have now established role in the treatment of patients with lung carcinomas harboring activating *EGFR* mutations. Compelling evidence to use *EGFR* genotyping to select patients to first-line EGFR inhibitor treatment come from the IPASS study [1] and from subsequent clinical trials that randomized patients with *EGFR* mutated tumors to EGFR inhibitor versus chemotherapy [2] [3] [4] [5]. Two large, placebo-controlled phase III trials compared erlotinib or gefitinib vs. placebo in the second or third line setting in unselected patients with advanced non-small cell lung cancer (NSCLC). Both studies indicated that the subset of patients harboring high *EGFR* gene copy number may derive significant benefit from EGFR inhibitor therapy [6] [7]. The cut-off point of *EGFR* positivity (defining “high gene copy number”) was previously determined as ≥4 *EGFR* copies in ≥40% of tumor cells, or numerous gene clusters observed in at least 10% of tumor cells [8]. With these background data, investigators at Central and East European Study Group (CEEOG) initiated the first-line multicenter, open-label, single arm, phase II trial (FLIKER), to evaluate the efficacy of EGFR TKI erlotinib in NSCLC patients with tumors harboring high *EGFR* gene copy number defined as above. This trial was commenced before a general adoption of *EGFR* mutations for selection of lung cancer patients to EGFR inhibitor treatment. We present here the final results of this trial, together with molecular analysis of *EGFR*, *KRAS* and *BRAF* mutation status in the tumor.

## PATIENTS AND METHODS

### Study design

The primary endpoint of this trial (CEEOG 0106, ML20033) was the proportion of patients alive and free of progression at 12 months after study entry. Secondary endpoints included response rate, overall survival, toxicity and feasibility of patient selection based on *EGFR* gene copy number.

Patients from seven Polish institutions collaborating within CEEOG were registered for molecular screening (*EGFR* gene copy number by FISH). Upon positive test performed centrally at the Medical University of Gdańsk, patients were included in the study and treated with erlotinib until disease progression, unacceptable toxicity or withdrawn consent. Patients with negative test were offered the best available treatment (most often chemotherapy and palliative radiotherapy) or best supportive care according to the decision of their primary physician.

High *EGFR* gene copy number was defined as ≥4 copies of the gene in ≥40% of tumor cells (high polysomy), presence of tight gene clusters, a gene-to-chromosome ratio per cell of ≥2, or ≥15 copies of *EGFR* per cell in ≥10% of analyzed cells (gene amplification). The protocols for *EGFR* gene copy number assessment, together with the definitions of “positive *EGFR* FISH test” were kindly shared for the purpose of this trial by dr Marileila Varella-Garcia, the Head of Cytogenetics Core Facility at the University of Colorado. All reagents and commercially available FISH probes (Abbott Molecular, Des Plaines, IL, USA) employed in FLIKER study, were used in accordance with the protocol developed at the University of Colorado. Before trial commencement, blinded set of slides received from the University of Colorado was scored at the Department of Biology and Genetics, Medical University of Gdańsk, to secure reproducible performance. Translational part of the trial included evaluation of *EGFR* FISH positive tumor samples for the presence of activating *EGFR, KRAS and BRAF* mutations with validated Cobas PCR-based, IVD certified tests (Roche Molecular Diagnostics, CA, Pleasanton, USA).

### Study subjects

Planed sample size of 72 subjects (53 events) was calculated assuming proportions of patients alive and free of progression at 12 months to be equal to 12.5% (P0, the null hypothesis) and 25% (P1, the alternative hypothesis implying the doubling of the proportion). With assumption of 32% of *EGFR* FISH positivity rate, 238 patients had to be screened with *EGFR* FISH. The trial was stopped prematurely on ethical grounds after 45 patients had been registered (149 screened for high *EGFR* copy number by FISH; 63% of planned sample size) due to convincing evidence to use *EGFR* mutations rather then copy number to select patients for first-line erlotinib treatment [1].

Patients with histologically confirmed stage IIIB (with pleural effusion) or stage IV NSCLC according to 6^th^ edition of TNM classification (all classified as stage IV according to current 7^th^ edition of TNM classification), with no prior systemic therapy for advanced disease, were prospectively enrolled on the study from seven CEEOG institutions in Poland. Paraffin-embedded tumor sample obtained from primary tumor or metastatic site at NSCLC diagnosis was required for analysis of *EGFR* gene copy number by FISH (cytology without specimen processing through cell block rendered the patient ineligible for the trial). Patients were required to have had clinically and/or radiographically documented measurable disease according to RECIST 1.0 criteria, to be >18 years old, to have WHO performance status of 0-2 and adequate hematological, hepatic and renal functions. No previous malignancies were allowed, except for adequately treated *in situ* carcinoma of the cervix or squamous carcinoma of the skin. Previous radiotherapy was acceptable, provided marker lesions were outside the irradiated volume.

The trial was approved by Local Ethics Committees of all participating institutions and the written informed consent had to be obtained before patient registration in the study.

### Drug administration

Continuous oral erlotinib (Tarceva, Roche, Switzerland) was given on an outpatient basis at a fixed dose of 150 mg taken at least one hour before or two hours after the ingestion of food or any other medication. In the event of toxicity (e.g. diarrhea, rash), regardless of its severity, that was not controlled by optimal supportive care or not tolerated due to any reason, the daily dose of erlotinib was decreased to 100 mg/day. Patients who could not tolerate the reduced erlotinib dose were permanently discontinued from the study.

Evaluation of response was performed according to the RECIST criteria. Target lesions were assessed by clinical examination and computed tomography (CT). The initial examinations had to be performed within 28 days prior to registration. Evaluation of target lesions was planned every four weeks for the first six cycles and then every eight weeks. Response had to be confirmed by second evaluation performed at least four weeks apart. Study visits for toxicity evaluation were scheduled every four weeks. Toxicity was assessed by Common Toxicity Criteria 3.0 of the National Cancer Institute. Quality of life or lung cancer symptom assessment was not performed in this trial.

### Statistical methods

Continuous variables were summarized by providing the number of observed values, mean, standard error, quartiles, and minimum and maximum values. Categorical variables were summarized by providing the counts and frequencies for the distinct categories. Survival curves were estimated with the use of Kaplan-Meier estimator. Confidence intervals for survival probabilities were constructed based on the log-log transformation of the survival function. Formal comparisons of survival curves were conducted using the log-rank test. The median follow-up time was estimated using the “reverse” Kaplan-Meier estimator, i.e., considering censored observations as failures and vice versa. Results of all statistical significance tests were assessed using the 5% significance level (two-sided). Computations were performed using SAS v. 9.3 and STATA v. 13.

## RESULTS

**Figure 1:**
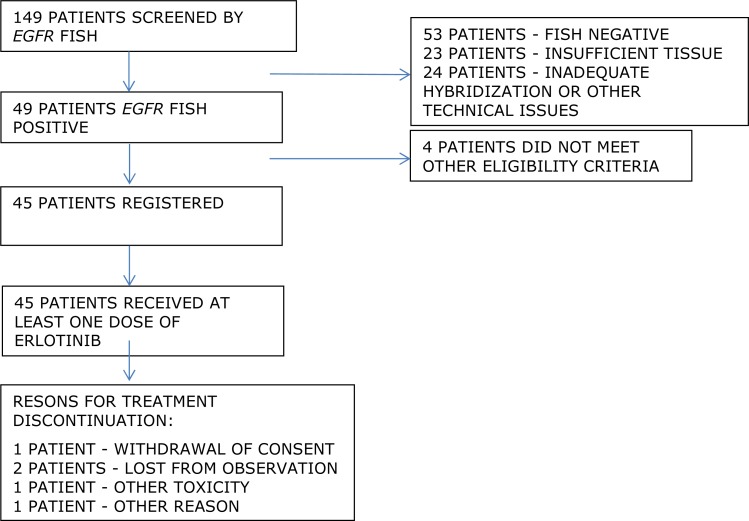
CONSORT diagram

Between December 2007 and April 2011, tumor samples from 149 patients were subjected to the molecular screening for the number of the *EGFR* gene copy number. *EGFR* FISH test was positive in 49 samples (33%) with the criteria described above, 53 samples (36%) were negative, 23 samples (15%) had an insufficient material and in 24 samples (16%) the hybridization was inadequate, precluding informative assessment. Out of 49 patients with positive *EGFR* FISH test on screening, 45 patients (37 with high *EGFR* polysomy and eight with *EGFR* amplification) were registered in the trial, and four did not meet predefined eligibility criteria. Consort diagram of the trial is provided in Figure 1.

The trial was stopped prematurely in April 2011 due to compelling evidence from other studies that NSCLC patients should be selected to first-line EGFR TKI therapy based on the presence of activating *EGFR* mutations, and that patients with tumors showing wild-type *EGFR* may be have inferior outcome when treated with first-line EGFR TKI as compared to chemotherapy.

Translational research part of the FLIKER trial included evaluation of samples from trial participants for the presence of *EGFR* mutations (*N* = 43, nine [21%] activating mutations detected), *K-RAS* mutations (*N* = 40, 11 [28%] mutations detected) and *BRAF* mutations (*N* = 30, no mutation detected). Decreasing number of samples analyzed for particular mutations was primarily due to insufficient or depleted material from tumors that had been previously subjected to other tests. *EGFR* mutations were found in 3 of 8 patients (38%) with true *EGFR* amplification and in 6 of 35 patients (12%) with no amplification (*p* = 0.33). *K-RAS* mutations were present in none of 7 (0%) and in 11 of 33 patients (33%) with or without amplification, respectively (*p* = 0.16).

Notably, study group included only five patients with squamous-cell carcinomas (11%), most patients had adenocarcinomas (*N* = 35, 78%; Table 1). The majority of patients (*N* = 35, 78%) had performance status of 0 or 1, and 10 patients (22%) had performance status of 2.

**Table 1:** Patient characteristics

	Characteristic	N	(%)
Age			
	Median (range) in years	66	(26-82)
Sex			
	Male	22	(49)
	Female	23	(51)
Performance Status			
	0	7	(16)
	1	28	(62)
	2	10	(22)
Histological type			
	Squamous-cell carcinoma	5	(11)
	Adenocarcinoma	35	(78)
	Large-cell	2	(4)
	NSCLC, NOS	3	(7)
Stage			
	IIIB	4	(9)
	IV	41	(91)
*EGFR* gene copy number			
	High-level polysomy	37	(82)
	Amplification	8	(18)
*EGFR* mutation			
	yes	9	(21)
	no	34	(79)
*KRAS* mutation			
	yes	11	(28)
	no	29	(72)
*BRAF* mutation			
	yes	0	(0)
	no	30	(100)

**Figure 2:**
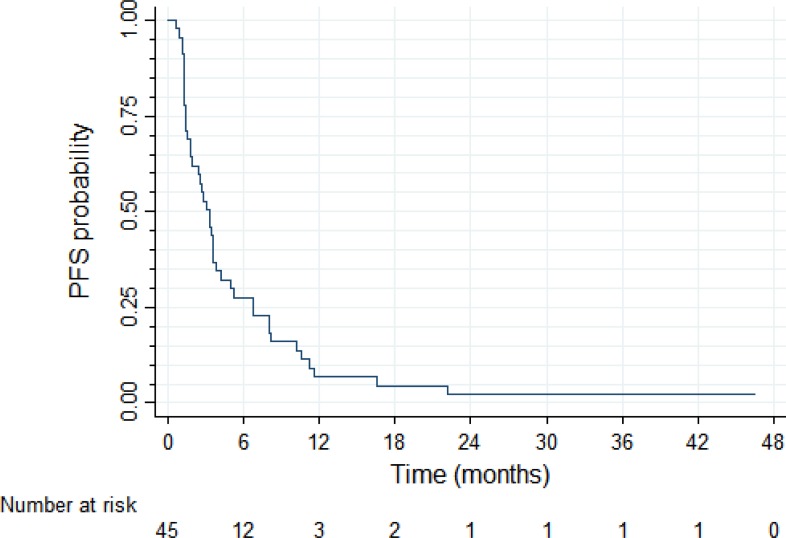
Progression-free survival (*N* = 45)

**Figure 3:**
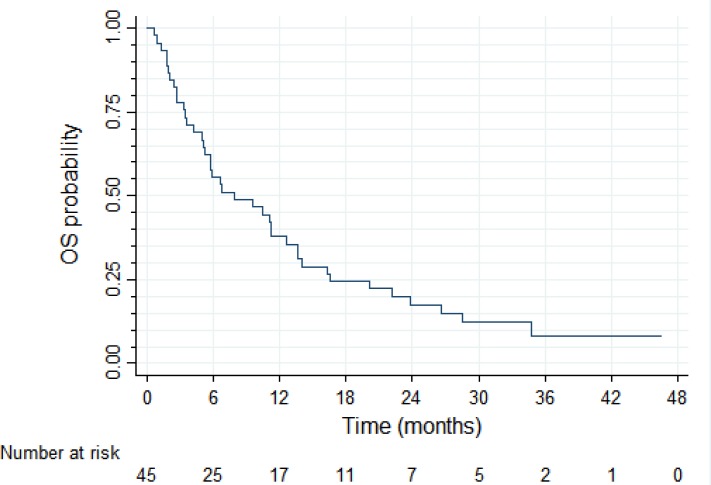
Overall survival (*N* = 45)

At the database lock (December 31, 2012) there were 43 PFS events among the 45 patients enrolled in the study. Four patients died without progression, 36 died with progression, three had progression and were alive, and two were alive and progression-free. After the median follow up time of 34.6 months, median PFS in the trial intent-to-treat (ITT) population was 3.3 months (95%CI: 1.8–3.9 months) and PFS probability at 12 months was 6.9% (95% CI: 1.8–17%; Figure 2). With 40 deaths among 45 patients, median overall survival was 7.9 months (95% CI: 5.1–12.6 months) and OS probability at 12 months was 38% (95%CI: 24–52%; Figure 3).

**Figure 4:**
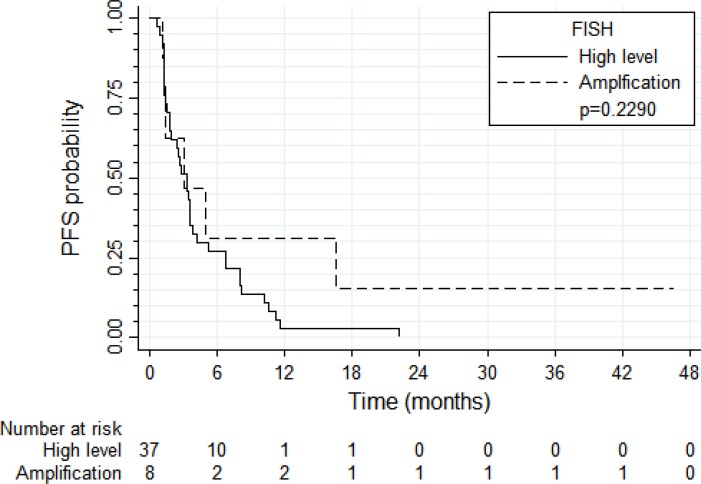
Progression-free survival according to *EGFR* FISH result

**Figure 5:**
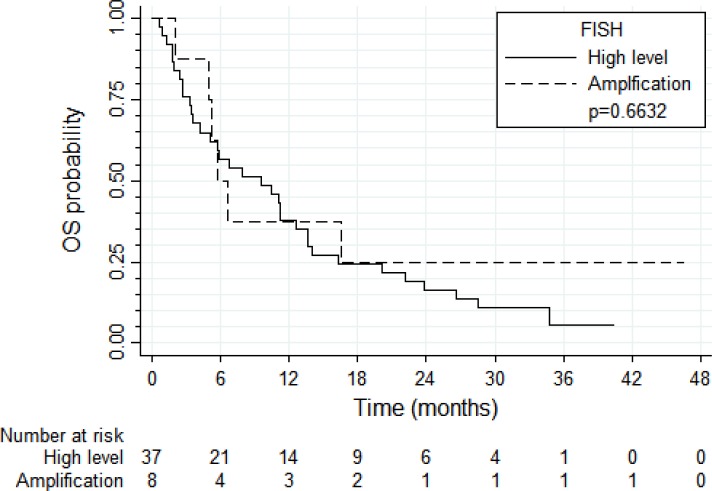
Overall survival according to the *EGFR* FISH result

**Figure 6:**
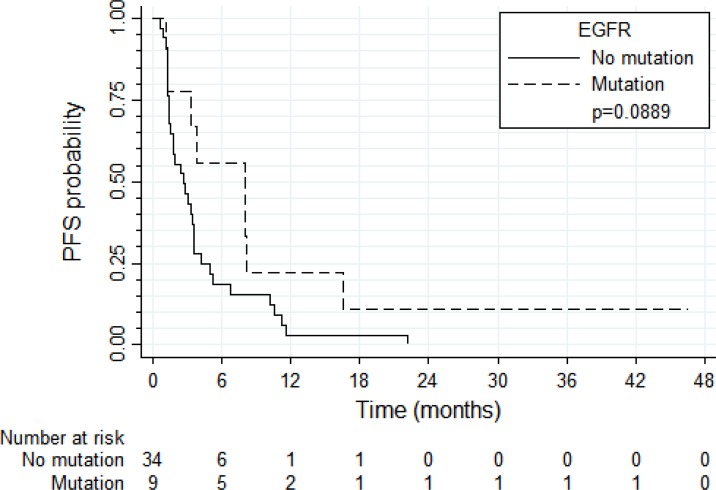
Progression-free survival according to the presence of *EGFR* mutations

**Figure 7:**
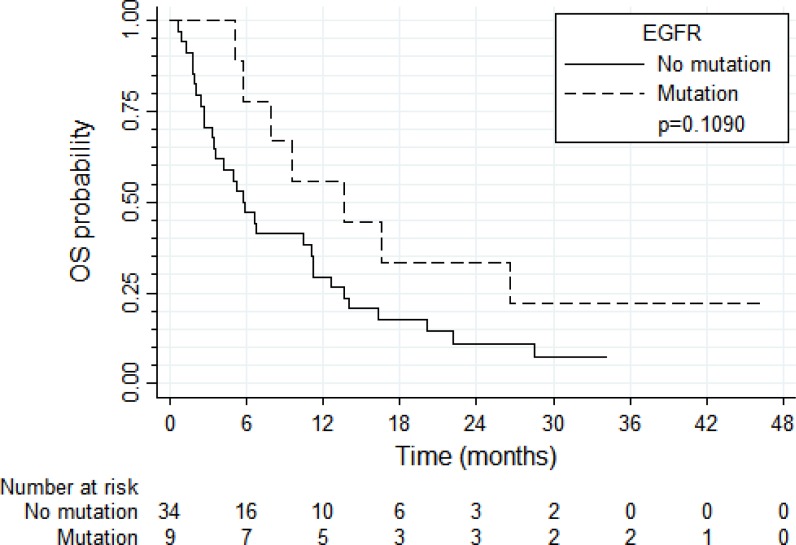
Overall survival according to the presence of *EGFR* mutations

PFS and OS results were broadly similar for 37 patients with tumors harboring high *EGFR* gene copy number vs. eight patients with *EGFR* gene amplification (N = 8) (Figure 4 and 5, respectively). Patients with tumors harboring *EGFR* mutations (*N* = 9) tended to have longer PFS than patients with wild-type *EGFR* (*N* = 36; median of 8.0 vs. 2.6 months, respectively, log-rank *P* = 0.089; Figure 6) and longer OS (median of 13.6 vs. 5.8 months, respectively, *P* = 0.109; Figure 7). Patients with tumors harboring *KRAS* mutations (N = 11) compared to those with wild-type *KRAS* (*N* = 29) tended to have shorter PFS (median of 1.4 vs. 3.5 months, respectively, log-rank *P* = 0.103; data not shown) with no significant difference for OS (median of 5.8 vs. 9.6 months, log-rank *P* = 0.91; data not shown).

Partial response was observed in five out of 45 patients (11%), stable disease in 13 patients (29%), progressive disease in 22 patients (49%) and five patients were not evaluable (11%). Response rates were similar in patients with high *EGFR* gene copy number (4/37 patients) vs. in those harboring *EGFR* gene amplification (1/8 patients). Responses appeared more common in patients with *EGFR* mutations (4/9 patients) compared to those with wild-type *EGFR* (0/34 patients). Responses were more common in patients with wild-type *KRAS* (4/29 patients) compared to those with tumors harboring *KRAS* mutations (0/11 patients).

Toxicity was evaluated using Common Toxicity Criteria 3.0. Adverse events were consistent with known erlotinib toxicity profile. Grade 3 toxicities were rare and included diarrhea (3/45 patients; 6.7%), skin rash (2/45 patients; 4.4%), dyspnea (1/45 patients; 2.2%), hiperbilirubinemia (1/45 patients; 2.2%), chest and spinal pain (2/45 patients; 4.4%) and hydropericardium (1/45 patients; 2.2%).

A total of 18 serious adverse events (SAEs), including seven events related to disease progression, were recorded in 17 trial participants. Six SAEs were related to erlotinib (unknown, doubtful or certain relationship to investigational product), including diarrhea (*N* = 1), diarrhea/vomiting (*N* = 1), exacerbation of chronic obstructive pulmonary disease (*N* = 2), dyspnea (*N* = 1) and fever/seizures (*N* = 1). None of these conditions led to protocol treatment interruption or withdrawal, and all improved or resolved during subsequent erlotinib therapy.

## DISCUSSION

We present here the results of prospectively designed trial evaluating the efficacy of erlotinib in advanced NSCLC patients selected by high *EGFR* gene copy number. The rate of *EGFR* FISH positivity in this trial (33%) compares well with reported series (31–38%) [6] [7] [9], and is in accordance with the protocol assumptions. The rate of *EGFR* activating mutations in this FISH-positive population was 21%, somewhat higher than in unselected NSCLC patients, but indicating a relatively weak relationship between both alterations. Increased *EGFR* gene copy number in *EGFR* mutant tumors have been demonstrated in other studies [6] [7]. The proportion of *EGFR*-mutated tumors in patients with true amplification was numerically higher compared to cases with high polysomy (38% and 12%, respectively), but a low number of patients with both *EGFR* mutations and *EGFR* amplification precluded a meaningful analysis. In the current study, no testing for the *EGFR* activating mutations was performed in patients with a FISH-negative status.

Although closed prematurely, the study provides convincing evidence against the use of increased *EGFR* gene copy number as a predictive marker for selection of NSCLC patients to first-line therapy with erlotinib. Median PFS of only 3.3 months compares unfavorably with 10-13 months observed with erlotinib in trials selecting patients by *EGFR* activating mutations [3] [4]. Our results are in line with other published evidence. In the IPASS study comparing gefitinib vs. carboplatin and paclitaxel, patients with *EGFR* mutated tumors had an apparent PFS advantage from gefitinib, and patients with wild-type *EGFR* from chemotherapy [1]. However, in patients with increased *EGFR* gene copy number without *EGFR* mutations, PFS with gefitinib was significantly shorter compared to chemotherapy (HR = 3.85) [10]. The results of this analysis prompted us to close FLIKER trial due to ethical concerns.

To the best of our knowledge this is the only prospective study using EGFR kinase inhibitor therapy, which enrolled exclusively patients with increased *EGFR* gene copy number. *EGFR* FISH positivity was also used to select patients to EGFR TKI inhibitor (gefitinib) in the ONCOBELL trial, but this study also included patients who never smoked or showed high phospho-Akt immunohistochemical staining[11]. In another trial comparing gefitinib and vinorelbine, the post-hoc exploratory analysis showed the superiority of chemotherapy in patients with high *EGFR* gene copy number [12]. Poor outcomes in patients with increased *EGFR* gene copy number (response rate of 13% and a median PFS of 8.4 weeks) were also found for afatinib, a second-generation EGFR TKI inhibitor [13].

The results of these studies, including ours, indicate that increased gene copy number does not seem to define oncogene-addicted tumors, as opposed to activating oncogenic mutations (e.g. *EGFR*, *HER2*, *BRAF*, *NTRK1*) or rearrangements (*ALK*, *ROS1* and *RET*). Perhaps selection of cut-off points at higher gene copy number levels might better define oncogene addiction and treatment benefit of targeting agents. In our study the subset of patients with germline *EGFR* amplification was too small to draw firm conclusion on this issue. However, such relationship has been demonstrated for other alterations. For example, out of three cohorts of NSCLC patients with increasing *MET/CEP7* ratios (1.8-2.2, 2.2–5.0 and above 5.0), the last had the highest tumor response rates to MET inhibitor crizotinib [14]. Therefore, clinical trials using gene copy number to select patients to targeted therapies should carefully define optimal cut-off levels and predictive values of “true amplifications”.

Our trial was based on results of biomarker data from two randomized, placebo-controlled trials (BR-21 and IRESSA) comparing respectively gefitinib and erlotinib vs. placebo in chemotherapy-pretreated NSCLC patients [15] [16]. Both trials demonstrated increased survival with TKI inhibitors in subjects with high *EGFR* gene copy number defined by FISH. The discrepant predictive value of *EGFR* copy number in chemotherapy-pretreated patients in these trials, and in chemotherapy naïve patients in our study is intriguing and warrants further investigation.

We are aware of limitations of this study, including small number of patients and closure of recruitment before the planned number of patients was reached. Nevertheless, our data do not support the use of *EGFR* gene copy number for prediction of benefit from first line erlotinib treatment in advanced NSCLC. Potential predictive value of gene amplification with high target gene levels merits further investigations.
